# Opportunities for successful de-escalation of proton pump inhibitors at a federally qualified health center

**DOI:** 10.1186/s40360-021-00486-x

**Published:** 2021-04-16

**Authors:** Joelle Ayoub, Jessina C. McGregor, Rebecca M. Castner, Harleen Singh

**Affiliations:** 1 Western University of Health Sciences College of Pharmacy, 309 E. Second St., Pomona, 91766 CA USA; 2 Oregon State University College of Pharmacy Portland Campus at Oregon Health & Science University, 2730 SW Moody Ave., CL5CP, Portland, 97239 OR USA; 3 Roosevelt University College of Pharmacy, 1400 N Roosevelt Blvd, Schaumburg, 60173 IL USA

**Keywords:** Proton pump inhibitors, Family medicine, Deprescribing, de-escalation, Underserved, Pharmacy

## Abstract

**Background:**

A Proton Pump Inhibitor (PPI) de-escalation initiative was piloted at a Family Medicine Federally Qualified Health Center (FQHC) after a needs assessment showed that PPIs were prescribed inappropriately. The objective was to evaluate implementation of a PPI de-escalation program for an urban, underinsured patient population at a (FQHC).

**Methods:**

Patients receiving PPI with an upcoming appointment with their primary care provider (PCP) were evaluated by a pharmacist for the appropriateness of therapy. The pharmacist administered a questionnaire to patients to assess PPI usage patterns and then evaluated for appropriate PPI therapy which included diagnoses, risk factors for gastrointestinal bleed, symptom control, and duration of PPI therapy. For consenting patients, de-escalation was implemented per pharmacist protocol.

**Results:**

A total of 36 patients were evaluated for appropriate PPI use, among those, 21 (58%) were eligible for de-escalation, and 19 agreed to de-escalation. Fifteen patients (15/19) had successful PPI de-escalation after 4 weeks without discomfort or symptoms which disrupted daily activities.

**Conclusions:**

This pharmacist led initiative in collaboration with PCPs resulted in successful de-escalation of PPIs in an underserved primary care setting.

**Supplementary Information:**

The online version contains supplementary material available at 10.1186/s40360-021-00486-x.

## Background

Proton pump inhibitors (PPIs) are the mainstay of therapy for management of acid-related disorders. PPIs are one of the most commonly prescribed medications in the United States with primary care physicians constituting the largest group of prescribers [1, 2]. However, it is estimated between 25 and 70% of the time these medications are used inappropriately either without an evidence-based indication or for longer durations than prescribed [1]. Both prescriber and patient factors have been identified as risk factors for inappropriate PPI use. PPIs are continued inappropriately due to lack of provider questioning the origin or reason for use [3]. This could be due to lack of time during visits, or low priority performing rigorous follow ups for the initial PPI indication. Yet, evidence suggests that inappropriate use can cause a wide variety of adverse effects including hip, wrist and spine fracture, *Clostridioides difficile*-associated diarrhea, community-acquired pneumonia, hypomagnesemia, chronic kidney disease and acute kidney injury [4]. In light of these safety concerns, several PPI stewardship programs have been implemented to discontinue or de-escalate inappropriate PPI therapy in both outpatient and inpatient settings [5].

Various PPI de-escalation strategies have been studied, but there is still a lack of consensus regarding the most appropriate approach for cessation of therapy [6]. In 2017, Canadian Family Physician published de-prescribing guidelines that recommend both abrupt discontinuations, tapering or using “on demand” PPI as viable options [7]. On demand PPI therapy is synonymous to a patient administering a PPI as needed when they are experiencing symptoms. The guidelines further emphasize that PPI de-prescribing should be a shared decision between the provider and the patient [7]. Use of histamine type 2 receptor antagonists (H2RAs) have also been used in de-escalation and are associated with less risk of pneumonia and chronic kidney disease progression/acute kidney injury in comparison PPIs [8]. While other long term side effects require further study when comparing PPIs to H2RAs, step down to a less potent acid suppressant such as H2RAs can be considered a significant de-escalation step, while achieving the goal of preventing acid relapse, with the ultimate goal to discontinue all acid-suppressing agents [6]. A recent review by Thompson et al. suggests patient attitudes should be incorporated into shared decision making [9]. Patient education, including the rationale for PPI de-escalation, as well as what to expect from de-prescribing, should be encouraged [9]. Further evidence also suggests shared decision making interventions significantly improve outcomes for disadvantaged patients with low literacy or socioeconomic status [10].

PPI prescribing patterns or de-prescribing initiatives have not been well studied in the underserved population. The high prevalence of mental health comorbidities, food insecurity, and substance abuse in the underserved population poses additional barriers to successfully sustain PPI de-escalation. A baseline needs assessment of a random sample of forty patients receiving a PPI was performed through a retrospective chart review to determine appropriate prescribing of PPIs at our FQHC. Appropriateness of therapy was based on indication and duration of PPI therapy. Duration of therapy exceeding 8 weeks for a diagnosis of GERD or dyspepsia was considered inappropriate. We identified that 12.5% of patients had no indication, and 67.5% received PPIs inappropriately for an extended duration of therapy (median duration of 628 days). This initial review revealed opportunities for intervention and led to a pharmacist-driven PPI de-escalation initiative.

### Aim of the study

Our objective was to evaluate implementation of a PPI de-escalation program for an urban, underinsured patient population at a federally qualified health center (FQHC).

## Methods

### Study setting and patient population

This project was conducted at a FQHC clinic affiliated with Oregon Health & Science University Department of Family Medicine. The clinic serves an urban, underinsured patient population of over 16,000 patients. The clinic is divided into four interprofessional teams; the core of these teams consists of one to three physicians, one to three nurse practitioners, zero to two physician assistants, three to four medical assistants, one to two nurses, one behavioral health practitioner, and two clinical pharmacists. Each team serves approximately 4000–5000 patients annually. These interprofessional teams share a collaborative workspace to facilitate patient care discussions throughout the day. The pharmacist assists with the management of chronic disease states under collaborative drug therapy management (CDTM) protocols, completes medication reconciliations post-hospital discharge, and provides drug information consultations. Within the clinic there is a 340B pharmacy which provides low-cost drug pricing.

#### Ethics approval

This quality improvement project was not deemed human subjects research by the IRB.

### Patient eligibility

**Fig. 1:**
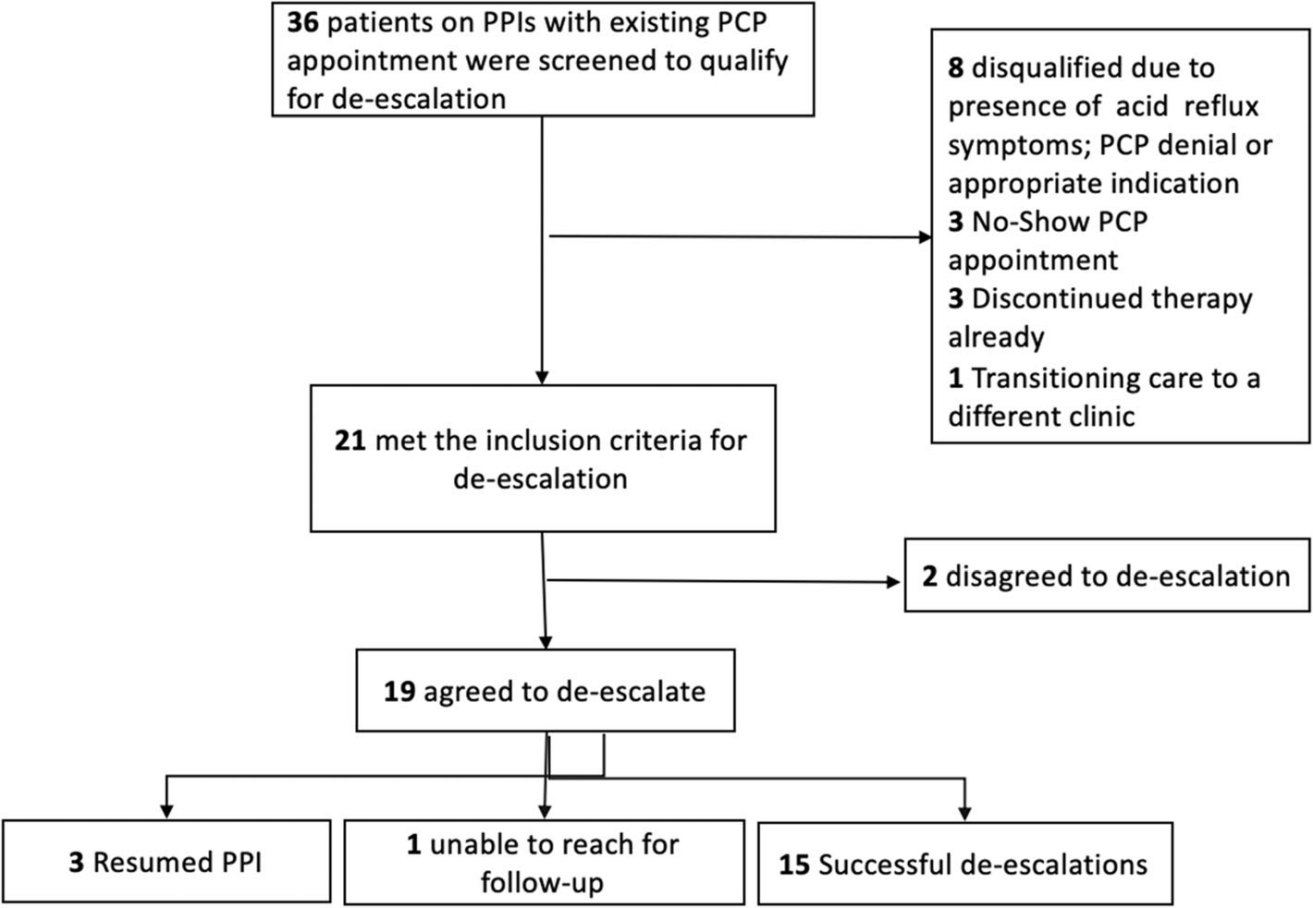
Stepwise Approach to De-escalation

Medical records for patients 18 years and older with an active PPI prescription in January 2019 were screened. Patients who were not followed by their PCP within the last year were excluded. We included patients from one interprofessional team to intervene. For patient convenience, only patients with an upcoming appointment with their PCP were reviewed by the pharmacist to implement the de-escalation process. An algorithm developed by Reeve et al. was used to determine patient eligibility for de-escalation [9]. Patients who had current symptoms of GERD, or with Barret’s esophagus, Zollinger Ellison syndrome, *H. Pylori* or utilized PPI for drug-induced/secondary peptic ulcer disease prevention were not eligible for de-escalation [11]. Those receiving PPI with no clear indication or those with inappropriate duration of therapy and not complaining of GERD or dyspepsia symptoms were considered eligible for de-escalation [10]. Additionally patients who were prescribed a PPI concomitantly with aspirin and nonsteroidal anti-inflammatory drug (NSAID) were evaluated to determine appropriate need for continuation of PPI therapy [10]. Patients who reported alarm symptoms or high symptom burden were referred to a gastroenterology specialist by PCP (Fig. 1).

### PPI de-escalation intervention

A CDTM protocol was developed prior to study initiation so that the pharmacist could prescribe appropriate therapy to facilitate de-escalation. For the purposes of this study, de-escalation is defined as any reduction in dose or frequency of use, or discontinuation of the PPI. Step-down therapy with a H2RA such as ranitidine is also defined as de-escalation [6]. PPI therapy was classified as low, standard or high dose as defined by National Institute for Health and Care Excellence (NICE) Guidelines for GERD management [12]. Low dose PPI therapy for GERD per NICE includes on demand omeprazole 10 mg, pantoprazole 20 mg, lansoprazole 15 mg once a day [11]. Standard dosing includes the next available dosage once daily (i.e. omeprazole 20 mg once daily), while double dose includes any dose or frequency beyond standard dosing (i.e. omeprazole 40 mg once daily.) [12]

A pharmacist initiated the initial assessment for PPI de-escalation and documented their assessment in the patient’s health record, prompting a PCP to review if de-escalation was appropriate [13]. In addition to electronic communication, PCPs were prompted to agree/disagree verbally with the pharmacist evaluation for appropriateness of PPI therapy. Refill history was reviewed to determine the duration of therapy and then verbally verified by the patient. Qualified patients for de-escalation were then scheduled to see the study pharmacist. PPI adherence and willingness to consider PPI de-escalation was verified by the medical assistant or the provider prior to meeting with the pharmacist.

The pharmacist met with the patient for 10–15 min to provide education on typical gastroesophageal reflux disease (GERD) symptoms, long-term adverse effects, and lifestyle modifications to prevent reoccurrence of symptoms as well as assess readiness for PPI de-escalation. A thirteen-item questionnaire (Appendix A) was administered to determine if the patient was ready and willing to taper their PPI based on symptoms and use. At the end of the visit, a handout (Appendix B) was distributed to patients to further re-enforce the key points regarding symptoms, lifestyle modifications, and PPI adverse effects. The handout was developed for low health literacy, and approved by the clinic’s health literacy committee.

**Fig. 2:**
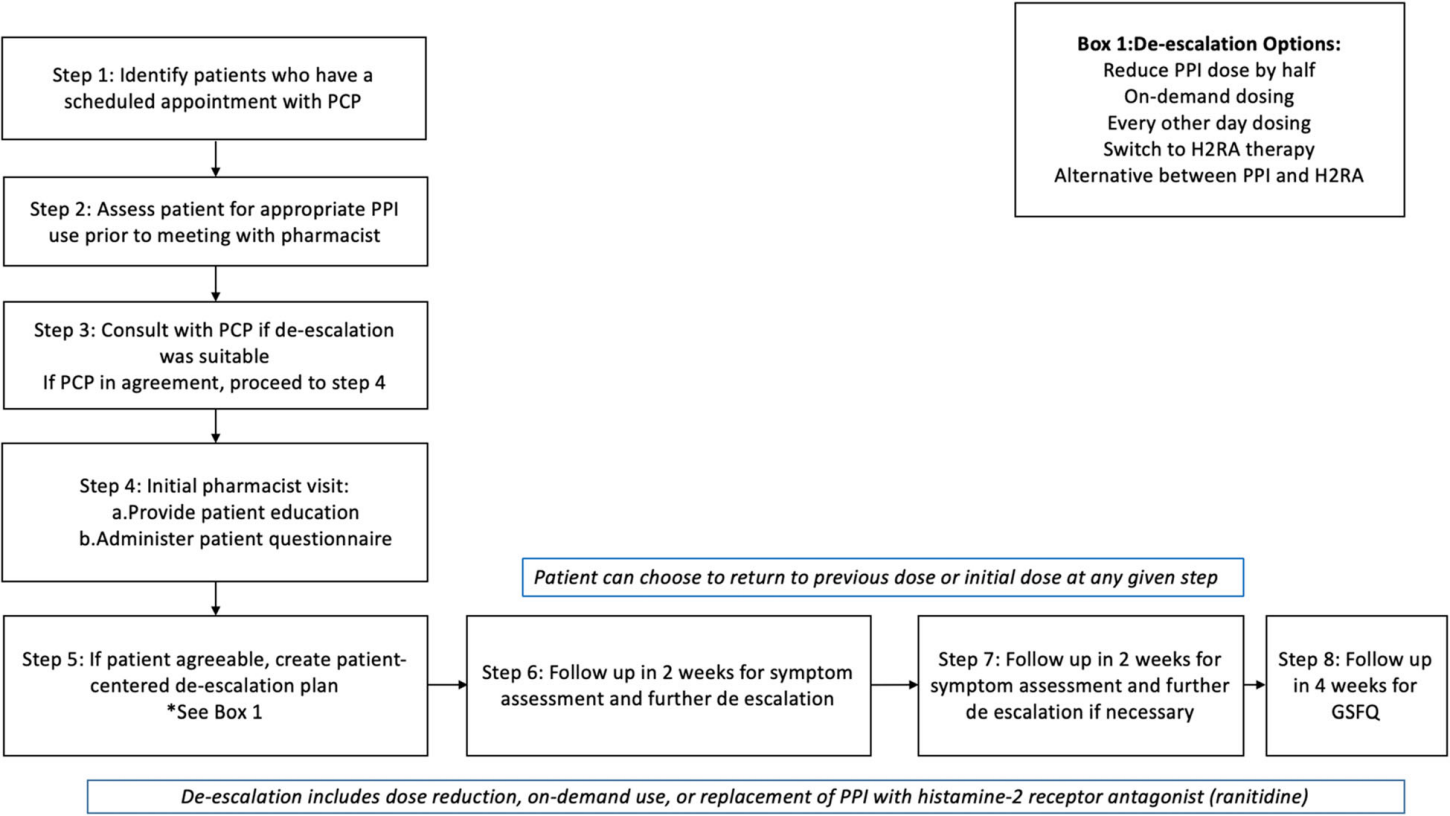
Selected Patient-specific De-escalation Scenarios

**Fig. 3:**
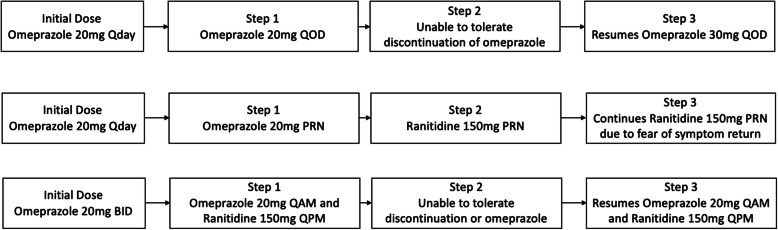
PPI De-escalation

For eligible and consenting patients, de-escalation was initiated using a modified version of the tapering algorithm developed by Bundeff et al. [13] De-escalation of PPIs were performed via a structured, stepwise protocol. Initially the de-escalation process highly relied on the algorithm created by Bundeff et al. [12] which provided a stepwise and individualized approach based on initial dose, frequency and tolerance of de-escalation. However, there were deviations from the algorithm depending on patient specific factors such as length of PPI therapy, convenience, and willingness of patient to proceed with de-escalation steps. (See Fig. 2 for specific examples). Patients were monitored every 2 weeks by clinic visit, phone call, or electronic health record messaging and followed for a total of 8 weeks. If symptoms occurred at any step, patients were instructed to return to the dose from the previous step or the initial dose prior to intervention if appropriate. De-escalation could be terminated at any step if the patient experienced acid reflux symptoms. The average time spent by the pharmacist on each patient was approximately 2 h. This included an hour for chart review and documentation to assess appropriateness of PPI use, 30 min for the initial face-to-face appointment with the patient and documentation, and 15 min per follow up phone call (Fig. 2 and 3).

### Assessment procedures

To ensure successful de-escalation and symptom frequency, the Gastrointestinal Short Form Questionnaire (GSFQ) was administered 4 weeks after the last de-escalation step [13]. This questionnaire assesses GERD symptom frequency; higher scores are associated with increased frequency and severity of symptoms.

#### Analyses

Patient characteristics were summarized by frequency and percent for categorical variables or median and interquartile range (IQR) for continuous variables. We calculated the proportion of patients with inappropriate PPI use as the proportion of patients with a lack of indication or excessive duration of therapy was calculated. The results of patient surveys were summarized to quantify adherence to PPI, over-the-counter (OTC) antacid or supplement use, food insecurity, and willingness to de-escalate.

## Results

Overall, a total of 958 patients had a PPI on their active medication list within the FQHC at the start of the study period. Within our selected study team we identified 234 patients who had a PPI listed on their medication list. Among these, 36 patients were reviewed for possible de-escalation who had an existing appointment with their PCP during the study period. The baseline characteristics for the 36 patients are as shown in Table 1. Median age of participants was 59, with median of 17 medications. Medicaid was the primary payer (72%), and patients were predominantly females (69%). Fifty percent of the patients were using PPI for GERD or heartburn and 33% of patients did not have an indication for PPI therapy. The most common PPI prescribed was omeprazole, with majority of patients receiving standard dose of therapy. Fifteen patients did not qualify for de-escalation for various reasons 47% had an appropriate indication or advanced reflux symptoms that required extended length of therapy. The remaining 21 of 36 patients (58%) qualified for de-escalation and 19 agreed to consider de-escalation. There were 15 successful de-escalations; 6 patients experienced return of symptoms at various steps but chose to continue de-escalation using H2RA for symptom control. The remaining patients’ de-escalation was tailored based on patient-specific factors. After 8 weeks and multiple de-escalation steps, eight patients remained on a lower dose or on demand PPI therapy, nine patients used H2RA as needed or scheduled, and two patients alternated between a PPI and an H2RA. Ranitidine was often used as a step-down therapy when patients complained of symptoms after tapering off the PPI. Of the 15 successful de-escalations, six patients were using H2RAs/PPIs only as needed/on demand by the end of de-escalation. All patients with successful de-escalation had a GSFQ score of 20 or less, indicating mild to moderate symptoms. Selected patient-specificde-escalation scenarios are highlighted in.

**Table 1:** Patient Demographics^a^

Characteristics	***N*** = 36
Median Age yrs. (IQR)	59 (42, 65)
Sex (female)	25 (69)
Insurer	
Medicare	6 (17)
Medicaid	21 (58)
Medicare/Medicaid	5 (72)
Commercial	4 (11)
Current PPI	
Omeprazole	28 (78)
Pantoprazole	5 (14)
Esomeprazole	1 (3)
Lansoprazole	2 (6)
Dose level	
Low	0 (0)
Standard	22 (61)
High	14 (39)
Median length of PPI use, days (IQR)	1160 (367, 1671)
Median medications (IQR)	17 (11, 32)
Median co-morbidities (IQR)	20 (13, 27)
Selected Co-morbidities	
Nutrient deficiencies	8 (22)
Tobacco dependence	11 (31)
Obesity	14 (39)
Mental Health	24 (67)
Indications	
GERD or heartburn diagnosis	18 (50)
No indication found	12 (33)

^a^Data are n (%) unless otherwise indicated. *IQR* Interquartile Range

The patient questionnaire was administered to all 21 patients who met study inclusion criteria and agreed to meet with the pharmacist. This questionnaire was used to evaluate PPI use, and revealed that 81% of patients were not aware of long-term adverse effects of PPIs and never received any formal patient education on adverse effects associated with PPI use or any discussion to consider discontinuation of therapy. Patients also reported infrequent use of OTC antacids or herbal supplements to treat acid related symptoms (4/21 patients), and a high level of effectiveness of PPIs (86%, 18/21). Most patients informed (19/21) that majority of their meals were home-made, and fast food was not routinely consumed. Furthermore, patients did not notify the pharmacist of any food insecurity. Out of 36 patients scheduled, 3 patients (8%) did not show up for their PCP appointments.

## Discussion

We identified opportunities to safely taper PPI therapy in patient who were receiving inappropriate therapy. Pharmacist collaboration with the PCPs facilitated development of patient-specific PPI tapering plans that were convenient and acceptable to patient. Our patients often use multiple sources of public transportation to attend PCP appointments, therefore we selected an approach that captured patients during their PCP visits; hence, patients did not have to incur additional travel costs to see the pharmacist. Furthermore, pharmacist communications with providers also increased PCP awareness to evaluate the appropriateness of PPI use during clinic visits which resulted in increased referrals for PPI de-escalation. Prior to this initiative, PPIs were prescribed with eleven refills without any assessment of symptom control between refills. Now providers prescribe PPIs for 8 weeks and schedule follow up with the pharmacist to assess efficacy of therapy.

Patients are often hesitant to discuss PPI therapy due the fear of reoccurrence of rebound symptoms. Once patients were educated regarding the long-term safety concerns with PPI use, they were more agreeable to de-escalate therapy, despite strong efficacy of PPIs and existing heartburn/GERD symptom control. Two of 21 patients refused de-escalation. Patients were also more willing to attempt de-escalation when step-down options were discussed. Typically, acid hypersecretion takes 3 months to resolve [14] however our patients tolerated de-escalation with minimal symptoms after the study period of 8 weeks [15]. Those with successful de-escalation did not experience any disruption in daily activities or inability to eat during the de-escalation process. Given our patient population is complex, commonly with psychiatric comorbidities and substance use disorders, it is likely that patients might restart PPI therapy in the future. Nevertheless, rigorous evaluations and continuous patient education will help to assess the long-term success of this intervention.

Generally, our patients do not feel comfortable seeking care from providers outside of the established care team. This was an anticipated barrier within our patient population; therefore we chose to recruit those with existing PCP appointments to establish trust via in person communication versus cold calls. The strength of the patient-provider relationship was critical to successfully engage patients in their health care. With PCPs seeing approximately 20–22 patients per day, each provider visit can only address one pertinent issue. PPI de-escalation requires time for education, counseling and creation of a personalized de-escalation approach, which PCPs did not have the bandwidth for. The study pharmacist, through the medical provider introducing the patient to the pharmacist in real-time and frequent phone follow-up, was able to build a trusting relationship with patients and the spend the time necessary.

The primary limitation of our study is the small sample size. Prior studies have reported de-escalation process to be challenging and were able to de-escalate only a small subset of patients despite beginning with a larger sample size [11, 13, 15, 16]. This was probably due to lack of provider buy-in or patient buy-in to de-escalate. Also, this process is time intensive and pharmacist may not have the dedicated time for such initiatives [11, 13, 15, 16]. Even though our sample size was small, a majority of the patients accepted the opportunity to de-escalate, and providers were supportive of this initiative. It is also possible that the electronic health records were unable to capture patients taking PPIs OTC, therefore we might have missed patients using OTC PPIs. However, we believe patients using OTC products for reflux symptoms will be low due to high cost of these medications and the low average income of our patient population. Additionally, the small number of patients may not have accurately represented the extent of food insecurity in this vulnerable population, therefore eating habits that would affect GERD and ability to control lifestyle interventions could be misrepresented. Another limitation was that the GSFQ was not administered at the beginning of the de-escalation, difference in symptom control pre and post de-escalation were not assessed [17]. Interestingly the GSFQ scores were low (0–20) and most patients reported no symptoms even after de-escalation, therefore scores prior to de-escalation may not have provided additional benefits. To our knowledge, this was the first study regarding PPI de-escalation in an underserved population. Given the paucity of data on PPI de-escalation in the underserved, our results have important clinical implications.

## Conclusions

PPI de-prescribing appeared to be well received by patients after educating the patient on lifestyle modifications and alternative therapies. Timely follow up and diligent monitoring is required for successful de-escalation. Warm handoffs assisted in creating strong patient relationships, which are especially important in this vulnerable population. Due to an increasing interest from providers in other teams throughout the clinic, this initiative will be expanded throughout the clinic by clinical pharmacists. Future opportunities for systematic PPI stewardship and patient recruitment include medication reconciliation by clinical pharmacists post hospital discharge, and medication counseling in the outpatient pharmacy located within the clinic. Expansion of this service is highly likely to improve appropriateness of PPI use and minimize PPI-associated adverse effects in this underserved population.

**Table:** 

JA	Contributed to conception and design	Contributed to analysis	Drafted the manuscript			Agrees to be accountable for all aspects of work ensuring integrity and accuracy
HS	Contributed to conception and design	Contributed to the interpretation		Critically revised the manuscript	Gave final approval	Agrees to be accountable for all aspects of work ensuring integrity and accuracy
JCM	Contributed to conception and design	Contributed to analysis		Critically revised the manuscript	Gave final approval	Agrees to be accountable for all aspects of work ensuring integrity and accuracy
RMC	Contributed to conception and design					Agrees to be accountable for all aspects of work ensuring integrity and accuracy

## Supplementary Information

**Additional file 1: Appendix A.** Patient Questionnaire

**Additional file 2: Appendix B.** Patient Education Handout

## Data Availability

The data that support the findings of this study are available from Oregon Health and Science University but restrictions apply to the availability of these data, which were used under license for the current study, and so are not publicly available.

## Abbreviations

PPI: Proton Pump Inhibitor
NSAID: Nonsteroidal anti-inflammatory drug
GERD: Gastroesophageal reflux disease
FQHC: Federally qualified health center
