# Supplementary material for: Opportunities for successful de-escalation of proton pump inhibitors at a federally qualified health center
Source: BMC Pharmacol Toxicol. 2021 Apr 16;22:20. doi: 10.1186/s40360-021-00486-x (PMC8052786; doi:10.1186/s40360-021-00486-x)
Supplement: Supplementary file 1 — Additional file 1: Appendix A. Patient Questionnaire [file 40360_2021_486_MOESM1_ESM.docx]

**Appendix A**: **Patient Questionnaire:**

1. What did your doctor tell you this was for?
   1. Heartburn
   2. Reflux
   3. I don’t know why I am taking this medication
2. If unknown, Are you using this medication for heartburn or acid reflux?
   1. Yes
   2. No
3. Is the medication called _______ (Confirm)
4. How many times a week do you take this medication?
   1. Less than once a week
   2. 1-2x a week
   3. 3-4x a week
   4. 5-6x a week
   5. Everyday
   6. I don’t know
5. Do these medications help with your symptoms?
   1. Yes for all symptoms
   2. Some symptoms
   3. Sometimes, not always
6. Has anyone recommended you stop the medication?
   1. Yes and I stopped and started again
   2. Yes and I refused
   3. No
7. Do you use anything over the counter to help with heartburn?
   1. Prevacid (lansoprazole)
   2. Nexium (esomeprazole)
   3. Prilosec (omeprazole)
   4. Zegarid (omeprazole)
   5. Tums (calcium carbonate)
   6. Maalox (Alum-Mag Hydroxide-Simeth)
   7. Gaviscon (Aluminum hydroxide and Magnesium carbonate)
   8. Zantac (ranitidine)
   9. Pepcid (famotidine)
   10. None
8. If yes, how many times a week do you use the over the counter medications to help with heartburn?
9. Do you use any home remedies for heartburn? If yes, which ones?
   1. Apple cider vinegar with water
   2. Lemon with water
   3. Manuka honey
   4. Licorice root
   5. Peppermint
   6. Chamomile
   7. Papaya enzyme
   8. Others
10. How frequently do you eat at a fast food restaurants such as a gas station, McDonalds, Carls Jr, Taco Bell, KFC, etc?
    1. Once a week
    2. 2-3x a week
    3. 3-4x a week
    4. Everyday
11. What percentage of the time do you eat home-cooked meals?
    1. 70-100%
    2. 50-70%
    3. <50%
12. Are you given any meals you do not have control over provided by a food bank?
    1. Yes
    2. No
13. This medication, when used for many years, can have some unwanted side effects. This medication brings down the acidity in your stomach, which can cause infections. The bacteria causing these infections are usually killed by the acidity in your stomach, but when your stomach is less acidic, they can cause pneumonia, or a stomach infection. They can also cause your body to absorb less nutrients like low vitamin B12 and magnesium, which can then lead to frail bones. Do you feel comfortable with slowly weening off this medication if your provider agrees, with the assistance of a pharmacist?
    1. Yes
    2. No
