# Supplementary figures and images for: Opportunities for successful de-escalation of proton pump inhibitors at a federally qualified health center
Source: BMC Pharmacol Toxicol. 2021 Apr 16;22:20. doi: 10.1186/s40360-021-00486-x (PMC8052786; doi:10.1186/s40360-021-00486-x)

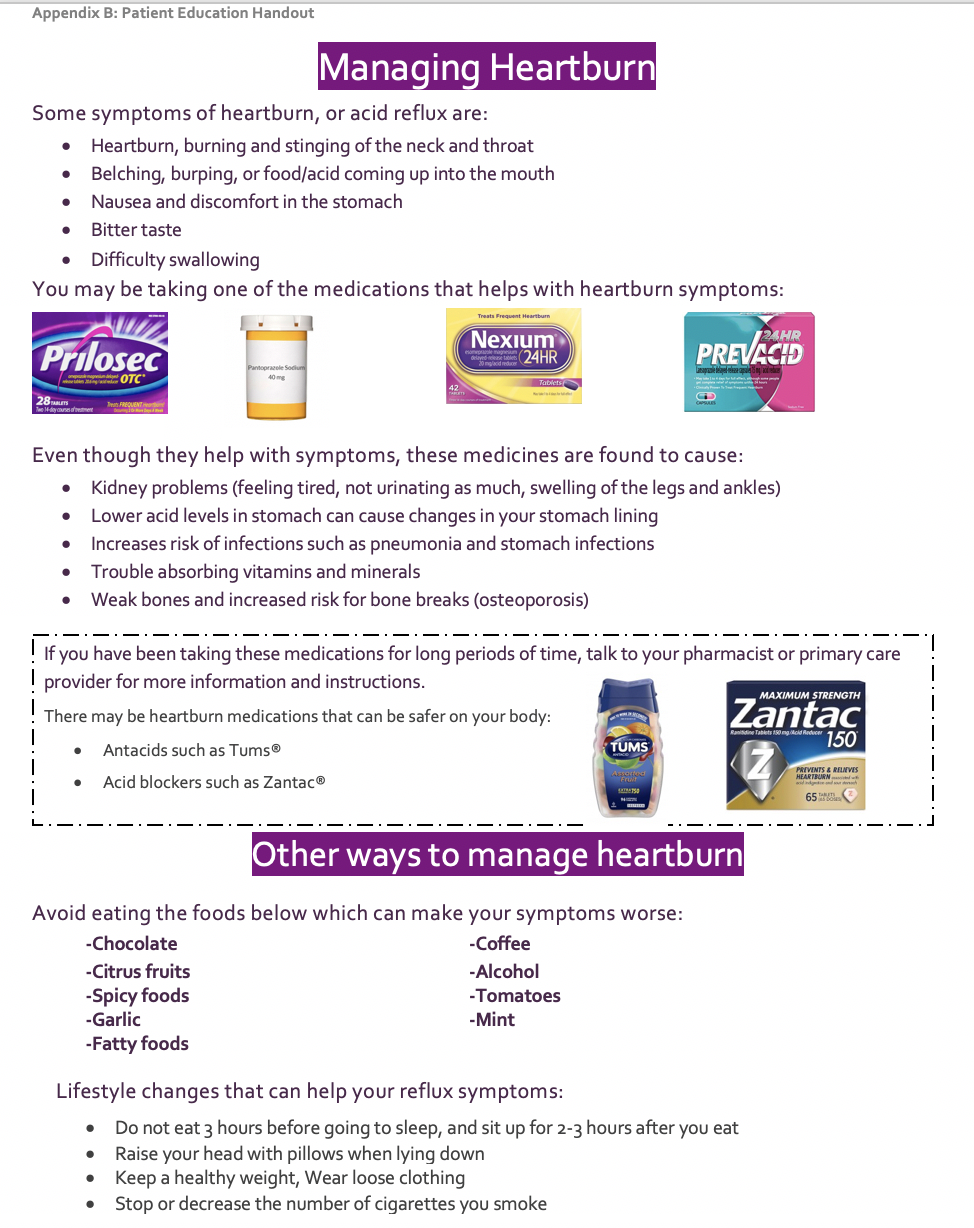

Supplement: Supplementary file 2 — Additional file 2: Appendix B. Patient Education Handout [file 40360_2021_486_MOESM2_ESM.docx]
